# Study of the l-Phenylalanine Ammonia-Lyase Penetration Kinetics and the Efficacy of Phenylalanine Catabolism Correction Using In Vitro Model Systems

**DOI:** 10.3390/pharmaceutics13030383

**Published:** 2021-03-13

**Authors:** Lyubov Dyshlyuk, Stanislav Sukhikh, Svetlana Noskova, Svetlana Ivanova, Alexander Prosekov, Olga Babich

**Affiliations:** 1 Natural Nutraceutical Biotesting Laboratory, Kemerovo State University, Krasnaya Street 6, 650043 Kemerovo, Russia; soldatovals1984@mail.ru; 2 Department of Bionanotechnology, Kemerovo State University, Krasnaya Street 6, 650043 Kemerovo, Russia; stas-asp@mail.ru; 3 Institute of Living Systems, Immanuel Kant Baltic Federal University, A. Nevskogo Street 14, 236016 Kaliningrad, Russia; svykrum@mail.ru (S.N.); olich.43@mail.ru (O.B.); 4 Department of General Mathematics and Informatics, Kemerovo State University, Krasnaya Street 6, 650043 Kemerovo, Russia; 5 Laboratory of Biocatalysis, Kemerovo State University, Krasnaya Street 6, 650043 Kemerovo, Russia; a.prosekov@inbox.ru

**Keywords:** phenylketonuria, l-phenylalanine ammonia-lyase, enzyme, kinetics, catabolism disorder, biomedical drug

## Abstract

The kinetics of l-phenylalanine ammonia-lyase (PAL) penetration into the monolayer of liver cells after its release from capsules was studied. The studies showed the absence of the effect of the capsule shell based on plant hydrocolloids on the absorption of l-phenylalanine ammonia-lyase in systems simulating the liver surface. After 120 min of incubation, in all variants of the experiment, from 87.0 to 96.8% of the enzyme penetrates the monolayer of liver cells. The combined analysis of the results concludes that the developed encapsulated form of l-phenylalanine ammonia-lyase is characterized by high efficiency in correcting the disturbed catabolism of phenylalanine in phenylketonuria, which is confirmed by the results of experiments carried out on in vitro model systems. PAL is approved for the treatment of adult patients with phenylketonuria. The encapsulated l-phenylalanine ammonia-lyase form can find therapeutic application in the phenylketonuria treatment after additional in vitro and in vivo studies, in particular, the study of preparation safety indicators. Furthermore, it demonstrated high efficacy in tumor regression and the treatment of tyrosine-related metabolic disorders such as tyrosinemia. Several therapeutically valuable metabolites biosynthesized by PAL via its catalytic action are included in food supplements, antimicrobial peptides, drugs, amino acids, and their derivatives. PAL, with improved pharmacodynamic and pharmacokinetic properties, is a highly effective medical drug.

## 1. Introduction

Among hereditary metabolic diseases, a separate group includes diseases associated with amino acid metabolism disorders. To date, about 90 inborn errors in amino acid metabolism (primary aminoacidopathies) are known, including phenylketonuria (PKU). Although phenylketonuria is considered a rare hereditary disease, it is an urgent public health problem for the Russian Federation and many other countries [1]. The incidence of phenylketonuria among children in the world averages one in 10,000 newborns and ranges from 1:200,000 (Thailand) to 1:4370 (Turkey). The higher the level of consanguinity in the population, the higher the incidence of genetic diseases, including phenylketonuria [2]. In Russia, according to neonatal screening data, the frequency of phenylketonuria averages 1:7000 and varies by region from 1:4735 in the Kursk region to 1:18,000 in the Tyva Republic. The most common form is the classic phenylketonuria, in which diet therapy is currently the only effective treatment method [3].

Research on the approaches to phenylketonuria treatment has been conducted quite actively for over 50 years [4]. Nevertheless, the main possibility for phenylketonuria patients to maintain health remains a rigid diet that limits the consumption of protein foods not only of animal but also of plant origin. According to William B. Hanley (2012), the cost of such food per person is 20–40 thousand dollars per year. Therefore, the major directions of research on this issue, carried out in many countries of the world (USA, Germany, Great Britain, Brazil, Bulgaria, India, China, Russia, and many others), are limited to the creation of special products and the reduction in the cost of technologies for their production [5].

There have been reports of alternative therapy methods for patients with phenylketonuria (pharmacological treatment, enzyme therapy, transplantation, and gene modification) in recent decades. However, they are not used in the Russian Federation due to the lack of developed and available technologies. The most promising treatment is the use of the enzyme l-phenylalanine ammonia-lyase (PAL), both in the form of injections and capsules/tablets, which breaks down phenylalanine to safe products. Biomarin Pharmaceutical Inc. obtained the most significant results as part of the US national program. Despite many years of research on this disease, Biomarin Pharmaceutical Inc. was the first to order a survey among PKU patients on their quality of life and their therapy development expectations [6]. The survey showed that most respondents are interested in the development of new drugs and treatments, giving preference to oral administration of pharmaceuticals with the option of not following a rigid diet. These results confirm that the problem has not yet been resolved and remains relevant [7].

There are patents in the open press describing methods of obtaining PAL by cultivating the yeast *Rhodotorula* and other microbial cells (US4757015, US4636466, EP0140714, EP0321488), PAL purification (JP60172282, JP58086082), stabilization (US5753487, EP0703788, WO/1995/000151), application (US7531341, US20070048855, US20020102712, US7537923), etc.

On the Russian market, the PAL enzyme preparation can be purchased only from foreign manufacturers: a commercial preparation (EC 4.3.1.5), Sigma-Aldrich (USA), cost about 800 euros/10 units (~290 g). BioMarin Pharmaceutical Inc., which began the third stage of PEG-PAL clinical trials (a pegylated preparation of recombinant l-phenylalanine ammonia-lyase), achieved significant results in the development of technologies for PAL production. If this drug enters the pharmaceutical market, the drug’s cost will limit its availability, especially when imported into other countries [8].

The therapeutic use of PAL is limited due to its proteolytic instability and immunogenicity. There are no proven technologies for purification and stabilization, as well as a stable form that guarantees the preservation of the enzyme before direct reaction with phenylalanine, especially in the acidic environment of the stomach [9,10].

Earlier, we obtained an encapsulated l-phenylalanine ammonia-lyase form, for which the degradation dynamics in model biological fluids (gastric and intestinal juices) [11] and storage stability [12] were studied. The main goal of PAL encapsulation in shells based on plant hydrocolloids is to stabilize it and ensure the possibility of subsequent therapeutic use.

This work aims to study the encapsulated l-phenylalanine ammonia-lyase penetration kinetics and the efficacy of phenylalanine catabolism correction using in vitro model systems.

## 2. Materials and Methods

### 2.1. Objects of Research

The object of this research was l-phenylalanine-ammonia-lyase (powder, activity from 1.5 to 5.0 U mg^−1^, density 1192 kg m^−3^, thermal conductivity 3.36 W (m K)^−1^), obtained from the pigment yeast cultivation [12]. Encapsulated PAL was obtained as described in [11], sample No. 6: the capsule shell contains 10.0 wt % carrageenan, 10.0 wt % agar–agar, 10.0 wt % carboxymethyl cellulose, 5.0 wt % glycerin, and 65.0 wt % water.

To study the kinetics of PAL (encapsulated and not encapsulated) penetration into the liver cell monolayer, human cell lines (hepatomas Huh-7 and monocytes THP-1) (All-Union (Russian) collection of cell cultures, Institute of Cytology of the Russian Academy of Sciences, St. Petersburg, Russia) were used.

Human monocyte suspension THP-1 was grown in RPMI-1640 medium (Dia-m, Moscow, Russia) containing 10% heat-inactivated bovine serum albumin (FBS), 1 mM sodium pyruvate, 0.05 mM 2-mercaptoethanol, penicillin (100 IU/mL) and streptomycin (100 μg/mL). Human hepatoma cells Huh-7 were grown in DMEM medium (Dia-m, Moscow, Russia) containing 10% FBS, penicillin (100 IU/mL), and streptomycin (100 μg/mL). In co-cultures, cells were grown in a mixed medium (1:1) in a trans-well, where the cells were separated by a porous membrane (pore size 3 μm, distance 1 mm). In co-cultures, Huh-7 cells were planted at an initial concentration of 75 × 10^4^ cells per well, cultured overnight, after which THP-1 cells (30 × 10^4^ cells per well) were added. All cells were cultured in Costar 12-well plates (Corning-Costar, Corning, CA, USA). After the monolayer formation, the cells were passaged using a Versene dissociating solution and 0.25% trypsin solution. Cells were passaged every 3–4 days. The differentiated cells were cultured for 20–30 passages.

To create an in vitro model of the liver plate, 6-well plates (Corning-Costar, Corning, CA, USA) were used, into which BD Falcon™ Cell culture inserts with a pore size of 0.4 μm and a surface area of 4.2 cm^2^ were inserted (BD Falcon™, Toronto, ON, Canada). The THP-1 and Huh-7 cell lines were removed from the surface of the culture plastic with Versene solutions and 0.25% trypsin solution with Hanks’ salts. After obtaining the THP-1 cell suspension, 1.5 mL of the cell suspension with a cytosis of 2 × 10^5^ cells/cm^2^ was passed into the apical compartment (AC) of the test system, which is membrane wells. The number of cells in the suspension was counted in a Goryaev chamber. Similarly, a suspension of Huh-7 cells in a volume of 1.5 mL with a cytosis of 5 × 10^5^ cells/cm^2^ was sub-cultured onto membrane inserts.

The 2.5 mL of culture medium was added to the basolateral compartment (BC) of the test systems, which was the wells of a 6-well plate. The culture medium in the apical compartment (AC) and BC was changed by gentle pipetting every 24–48 h.

Model media simulating blood serum was used to analyze the efficiency of correction of disturbed phenylalanine catabolism by the developed form [11,12] of encapsulated l-phenylalanine ammonia-lyase on in vitro model systems. The model solution contained 5.5 mg bovine serum albumin, BSA (molecular weight 68,000 g/mol, isoelectric point 4.9), and 1.6 mg γ-globulin (molecular weight 200,000 g/mol, isoelectric point 6.0). The stock solution contained 1000 μmol/L of phenylalanine. Three series of experiments were carried out. In the first series, phenylalanine hydroxylase (PAH, number in the numerical classification of enzymes based on the chemical oxidation reactions they catalyze: EC 1.14.16.1), as well as tetrahydrobiopterin and ferrous salt, were added to the stock model solution. In the second series, phenylalanine transaminase (PAH, number in the numerical classification of enzymes based on the chemical transfer reactions of functional groups they catalyze: EC 2.6.1.58) was added to the stock model solution, in the third series—l-phenylalanine ammonia-lyase in the encapsulated form [11,12]. The enzyme activity varied in the range from 1.5 to 5.0 U/mg. All solutions were incubated for 4 h at 37 °C, taking samples every 30 min and recording the content of such substances as phenylalanine, tyrosine, trans-cinnamic acid, phenylpyruvate, phenyl lactate, and phenylacetate.

### 2.2. Determination of the Kinetics of PAL Penetration into the Monolayer of Liver Cells

Test systems in the form of membrane wells with 1.5 mL of cell suspension with a cytosis of 2 × 10^5^ cells/cm^2^ were preliminarily prepared. For this purpose, the cells were sub-cultured onto membrane inserts BD Falcon™ Cell culture inserts (Thermo fisher, Moscow, Russia).

Working concentrations of encapsulated [11,12] and not encapsulated l-phenylalanine ammonia-lyase (25, 50, and 100 mg) were prepared in Hanks’ salt solution immediately before the experiments. The working concentrations of the solutions were selected based on the experiments carried out to determine the PAL hepatotoxicity. After complete dissolution, the working solutions were sterilized in a cleanroom before being added to the cells using MS PES Syringe Filters (Membran solutions, Tokyo, Japan) with 0.22 μm pore diameter syringe filters.

To assess the interaction of capsules with the test preparation, the PAL solution was incubated with a solution prepared from crushed capsules after their complete dissolution. Further, the obtained values of the kinetics of penetration through the monolayer of cells were compared with the kinetics of penetration of the enzyme without added capsule solution.

The apical and basolateral compartments were washed twice with Hanks’ salt solution. Then, 2.5 mL of salt solution was added to the basolateral compartment and 1.5 mL of working PAL solutions to the apical compartment to determine the kinetics of drug penetration in the absence of capsules, or 750 μL of twice the concentration of PAL solution and 750 μL of salt solution to determine the kinetics of drug penetration in the presence of capsules.

The kinetics of encapsulated [11,12] and not encapsulated PAL penetration through monolayers of cells was assessed after 60 and 90 min, for which Hanks’ salt solution was aspirated from BC and AC with the further determination of the enzyme concentration in solutions. The l-phenylalanine ammonia-lyase concentration was determined spectrophotometrically at a wavelength of 280 nm.

### 2.3. Quantification of Phenylalanine and Tyrosine in Model Solutions

In model solutions simulating blood serum, the amount of phenylalanine and tyrosine was determined by HPLC using an LC-20 liquid chromatograph (Shimadzu, Kyoto, Japan). The method involves deproteinization of a small volume (300 μL) of a solution simulating blood serum, followed by HPLC on a PEEK cation exchange column (lithium form, 9 μm, 46 × 250 mm). We used the reaction of amino acids with ninhydrin with the formation of colored compounds, which were recorded on a UV detector at 570 nm. The flow rate was 25 mL/h in a gradient of six mobile phases (pH 2.80, pH 3.00, pH 3.15, pH 3.50, pH 3.55, LiOH); the duration of each analysis was 170 min. 

### 2.4. Quantification of Trans-Cinnamic Acid

The quantitative content of trans-cinnamic acid was determined by capillary electrophoresis on a Kapel-105 device (Lumex, St. Petersburg, Russia). Conditions for the electrophoretic determination of cinnamic acid: working electrolyte—10 mM Na_2_B_4_0_7_ solution containing 40 mM sodium dodecyl sulfate; sample injection 5 s, 30 mbar; voltage +20 kV; the detection wavelength is 205 nm [13].

### 2.5. Quantification of Phenylpyruvate, Phenyllactate, and Phenylacetate

The quantitative content of phenylpyruvate, phenyl lactate, and phenylacetate was determined spectrophotometrically based on the ability of these compounds to form complex colored compounds with ferric iron ions.

## 3. Results

**Figure 1:**
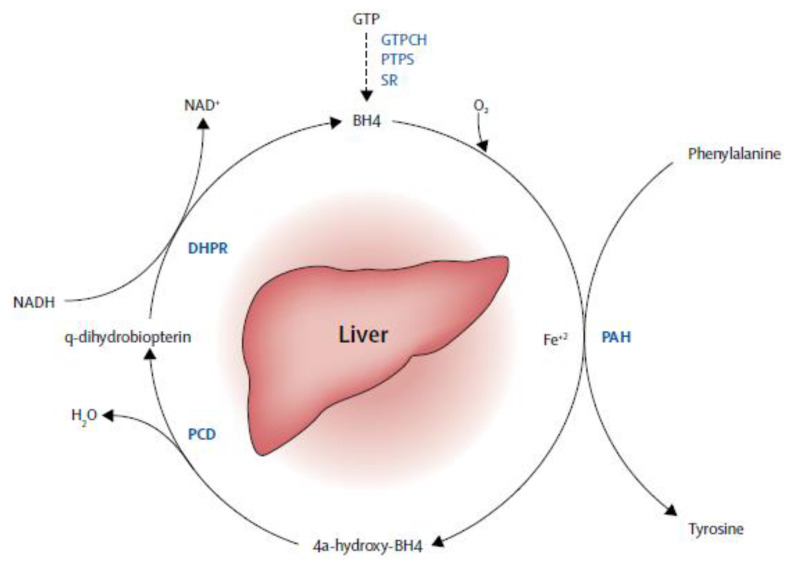
Phenylalanine hydroxylation system.

**Figure 2:**
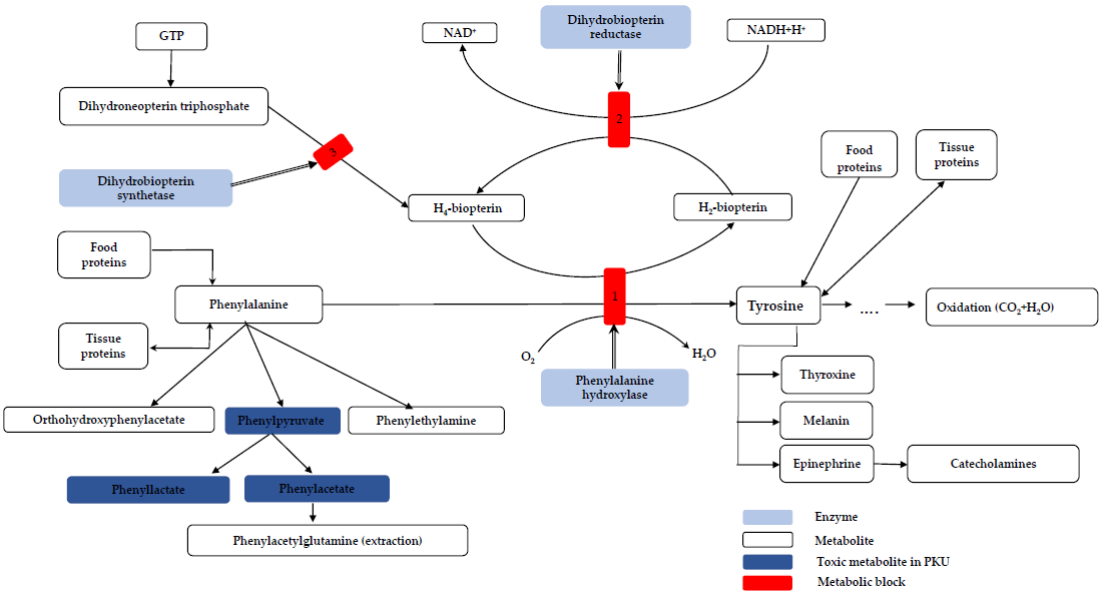
Scheme of metabolic processes that determine the mechanism of phenylketonuria development [14]: metabolic block 1—in case of phenylalanine hydroxylase deficiency (classical phenylketonuria); metabolic blocks 2–3—in case of various forms of BH_4_ deficiency (phenylalanine hydroxylase-independent hyperphenylalaninemia).

The phenylalanine hydroxylation system is shown in Figure 1; the scheme of metabolic processes that determine the development mechanism of PKU and some hyperphenylalaninemias in the case of BH_4_ deficiency is presented in Figure 2.

In BH_4_-dependent PKU forms, the metabolic block does not extend to the phenylalanine hydroxylation process itself but one of the stages of biosynthesis and regeneration of the active form of tetrahydrobiopterin [15].

**Scheme 1:**
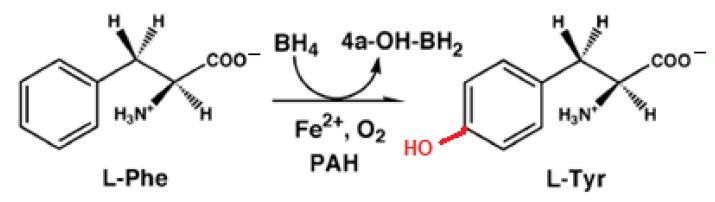
PAH-catalyzed reaction.

PAH is a non-heme, homotetrameric, iron-containing enzyme that requires BH_4_, molecular oxygen, and an active site-bound Fe^2+^ ion to convert phenylalanine to tyrosine (Scheme 1).

This enzyme plays an essential role in most catabolism reactions of phenylalanine from food and is localized mainly in the liver [12].

**Scheme 2:**
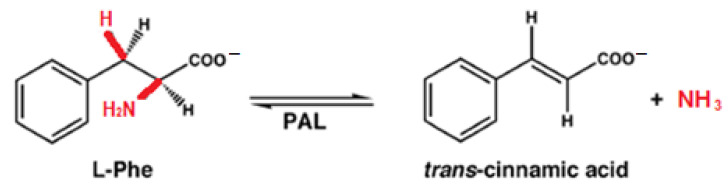
PAL-catalyzed reaction.

One of the promising PKU treatments is enzyme replacement therapy using two enzyme systems: PAH and l-phenylalanine ammonia-lyase (PAL). Compared to PAH, PAL therapy has many advantages. PAL does not require cofactors for phenylalanine degradation, and the reaction product, trans-cinnamic acid (Scheme 2), has low toxicity.

The calculated data on the kinetics of the encapsulated [11,12] and not encapsulated l-phenylalanine ammonia-lyase penetration into the monolayer of liver cells (cell line Huh-7, cell line THP-1, and co-culture Huh-7 + THP-1) at incubation times of 60, 90, and 120 min are presented in Table 1.

**Figure 3:**
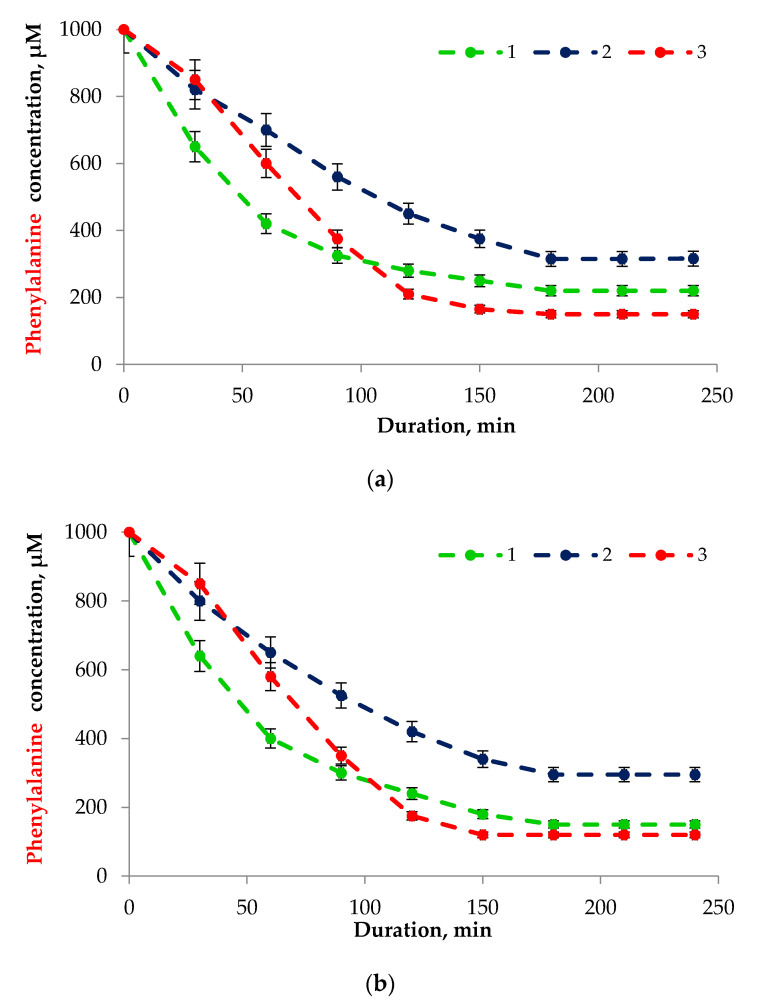

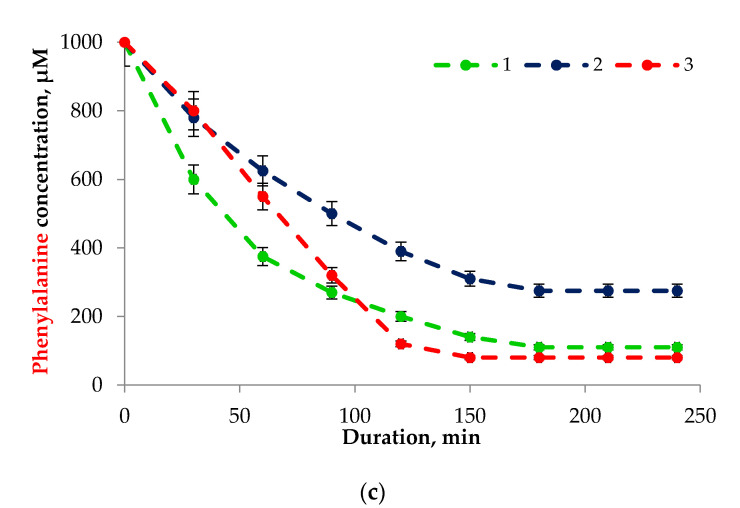
The phenylalanine concentration dynamics in a model medium (imitating blood serum) containing enzymes with activity (**a**) 1.5 U/mg; (**b**) 3.0 U/mg; (**c**) 5.0 U/mg depending on incubation duration: 1—PAH; 2—phenylalanine transaminase; 3—encapsulated l-phenylalanine ammonia-lyase. The experiments were carried out in triplicate.

The results of determining the phenylalanine concentration dynamics in model solutions of three series are shown in Figure 3.

The results of determining the content of phenylalanine metabolism products after incubation of model solutions with enzymes are presented in Table 2.

## 4. Discussion

Phenylketonuria is a genetic autosomal recessive disease. Classic phenylketonuria occurs due to mutations in the q22–24 region of chromosome 12, which affects the structure and function of phenylalanine hydroxylase. This enzyme is responsible for converting phenylalanine (Phe) to tyrosine (Tyr) in the liver. The decreased activity of phenylalanine hydroxylase, as a rule, causes phenylalanine accumulation in the blood and body tissues, including the central nervous system. The exact mechanism of the increase in phenylalanine levels, which can damage the brain, is still unclear. The same can be said about direct damage and a decrease in tyrosine levels, as well as a lack of other large neutral amino acids that interfere with neurotransmitter formation and neuronal development [16,17,18].

From the perspective of the pathogenetic links underlying hyperphenylalaninemia development, several forms of this pathology are distinguished. A classic PKU caused by several mutations in the phenylalanine hydroxylase gene, the general result of which is a deficiency of the enzyme activity [17,19,20], and PKU forms, previously called atypical, which are associated with impaired metabolism of tetrahydrobiopterin (BH_4_), a coenzyme involved in the hydroxylation of several amino acids, including phenylalanine [21,22].

In classic PKU, a defect in the phenylalanine 4-hydroxylase activity leads to a metabolic block in the conversion of phenylalanine to tyrosine, which results in abnormal metabolic product accumulation in the patient’s body in large quantities—phenylpyruvate, phenyllactate, and phenylacetate, the content of which is insignificant under normal conditions. These metabolites can penetrate the blood–brain barrier and exert neurotoxic effects, especially on the developing central nervous system of a child. It is assumed that they contribute to the disruption of the myelination processes of nerve fibers [23].

Since the main reactions of phenylalanine metabolism are happening in the liver, the study of the kinetics of l-phenylalanine ammonia-lyase penetration into the monolayer of liver cells after its release from capsules [11,12] is of considerable interest in this study.

In recent years, various in vitro models have been developed for performing experiments on liver cells. In this regard, three main, fundamentally different approaches can be distinguished: precision-cut liver tissue slice, cell cultures, and isolated perfused liver model. The advantage of using precision-cut liver slices is the ability to compare the analysis of cell and tissue morphology. Liver perfusion allows a range of physiological and morphological parameters to be assessed (tissue histology). Cell culture models can be effectively used to assess cell metabolism, cytotoxicity, and genotoxicity. However, such studies are complicated by the difficulty of maintaining the culture of hepatocytes [11,24,25].

Edling et al. proposed to use a system based on connecting human cell lines: hepatoma (Xa-7) and monocytes (THP-1) [15]. With the introduction of various substances, an increase in the expression of genes for anti-inflammatory mediators and genes associated with stress was observed in both types of cell.

In this work, human cell lines (Huh-7 hepatomas and THP-1 monocytes) were used to study the kinetics of encapsulated [11,12] and not encapsulated PAL penetration into the monolayer of liver cells. 

The results presented in Table 1 indicate that the kinetics of PAL penetration into the cell monolayer in the three test systems under study are practically the same. Moreover, it follows from Table 1 that the presence of a capsule shell based on plant hydrocolloids does not significantly affect the absorption of l-phenylalanine ammonia-lyase in systems simulating hepatic laminae.

Analysis of Figure 3 indicates that in all the studied model systems, there is a decrease in the concentration of phenylalanine with an increase in the duration of incubation, and the decrease in the concentration of this amino acid is directly proportional to the activity of enzymes: phenylalanine hydroxylase, phenylalanine transaminase, and encapsulated l-phenylalanine ammonium lyase. The minimum content of phenylalanine was recorded in a simulated blood serum containing encapsulated PAL: from 80 µmol/L to 150 µmol/L after 4-h incubation. The maximum content of phenylalanine was found in the model solution containing phenylalanine transaminase: from 275 μmol/L to 316 μmol/L after 4-h incubation with the enzyme.

A detailed analysis of Figure 3a allows us to conclude that the concentration of phenylalanine in a model medium (simulating blood serum) containing enzymes with an activity of 1.5 U/mg decreases with an increase in the incubation duration. The most significant decrease in phenylalanine concentration is observed for PAH, the smallest—for phenylalanine transaminase.

Analysis of Figure 3b allows us to conclude that the concentration of phenylalanine in a model medium (imitating blood serum) containing enzymes with an activity of 3.0 U/mg also decreases with an increase in the incubation duration. The most significant phenylalanine concentration decrease is observed for PAH, and to lower concentrations than in Figure 3b, the smallest—for phenylalanine transaminase.

Analysis of Figure 3c allows us to conclude that the concentration of phenylalanine in a model medium (imitating blood serum) containing enzymes with an activity of 5.0 U/mg also decreases with an increase in the incubation duration. The greatest decrease in phenylalanine concentration is observed for PAH, and to the lowest concentrations of the three presented enzymes. The smallest decrease in concentration is observed for phenylalanine transaminase.

Analyzing the results presented in Table 2, we concluded that the patterns of phenylalanine catabolism in biological fluids under the action of such enzymatic systems as phenylalanine hydroxylases, phenylalanine transaminases, and l-phenylalanine ammonia-lyase are consistent with theoretical data. Thus, upon incubation of a model medium simulating blood serum with PAH, an accumulation of tyrosine in the medium was observed: after incubation for 4 h at an enzyme activity of 5.0 U/mg, the concentration of this amino acid was 118.7 μmol/L. Thus, the phenylalanine metabolism proceeds according to the scheme shown in Scheme 1.

We found [12] that PAL stability during storage is longer in the encapsulated form than in the un-encapsulated one. By the 4th month of storage, the PAL activity in the capsule is 25.4% higher than in the absence of the capsule. Furthermore, by the 6th month, the activity of PAL in the encapsulated form is 46.4% higher than in the un-encapsulated one. Thus, it can be concluded that PAL is stable in encapsulated form for 6 months, which proves its superiority compared to un-encapsulated.

In the presence of the phenylalanine transaminase enzyme in the model environment, the accumulation of such products as phenylpyruvate, phenyllactate, and phenylacetate (toxic products of phenylalanine catabolism) is noted. These processes are shown in Figure 2.

Finally, in the model medium containing the enzyme l-phenylalanine ammonia-lyase (encapsulated form), the accumulation of trans-cinnamic acid is observed, the concentration of which after a 4-h exposure at a PAL activity of 5.0 U/mg is 138.2 μmol/L. The obtained data indicate that phenylalanine undergoes degradation according to the scheme shown in Scheme 2.

The work [26] presents the results of studies of the accumulation of tyrosine, phenols, and cinnamic acid in the medium at enzyme activity 1.0, 2.0, 3.0, 4.0, 5.0, 6.0, and 8.0 U/mg during incubation for 1, 2, 4, and 8 h. It was found that the concentrations of tyrosine, phenols, and cinnamic acid were the highest at the activity of l-phenylalanine ammonia-lyase 5.0 U/mg after incubation for 4 h. In [27], the PAL activity in relation to the accumulation of trans-cinnamic acid, tyrosine, flavonoids, benzoids, and phenolic glycosides was studied. The results obtained in [27] are in good agreement with the data obtained in our studies.

## 5. Conclusions

l-phenylalanine ammonia-lyase has recently become an important therapeutic enzyme with several biomedical properties [25]. The enzyme catabolizes l-phenylalanine to transcinnamate and ammonia. PAL is widespread in higher plants, some algae, ferns, and microorganisms, but is absent in animals. Although microbial PAL has been widely used in the past to produce industrially important metabolites, its high substrate specificity and catalytic efficiency have recently stimulated interest in its biomedical applications. PAL is approved for the treatment of adult phenylketonuria patients. In addition, it showed high efficacy in tumor regression and the treatment of tyrosine-related metabolic disorders such as tyrosinemia. The encapsulated l-phenylalanine ammonia-lyase form can find therapeutic application in the phenylketonuria treatment after additional in vitro and in vivo studies, in particular, the study of preparation safety indicators. This is evidenced by the results of studying the kinetics of encapsulated PAL penetration into a monolayer of liver cells, as well as the results of evaluating the effectiveness of capsules with PAL in terms of correcting impaired phenylalanine catabolism in phenylketonuria. Another important advantage of using l-phenylalanine ammonia-lyase in the capsule form is its stability during storage compared to the un-encapsulated enzyme [12] and the stability of the encapsulated preparation in the acidic environment of the stomach [11], which allows PAL to be released mainly in the intestine. Several therapeutically valuable metabolites biosynthesized due to their catalytic action are included in food supplements, antimicrobial peptides, drugs, amino acids, and their derivatives. PAL with improved pharmacodynamic and pharmacokinetic properties is a highly effective biological agent.

## Figures and Tables

**Table 1:** PAL content (%) in the basolateral compartment of test systems based on Huh-7 and THP-1 cell monolayers.

Cell Line	Incubation Conditions	Incubation Duration, min		
		60	90	120
The initial concentration of PAL 25 mg				
I	A	35.6 ± 1.8	68.9 ± 3.4	94.5 ± 4.7
	B	37.2 ± 1.9	67.3 ± 3.4	93.8 ± 4.7
II	A	41.4 ± 2.1	72.0 ± 3.6	95.2 ± 4.8
	B	39.8 ± 2.0	70.5 ± 3.5	95.0 ± 4.8
III	A	38.7 ± 1.9	69.5 ± 3.5	93.5 ± 4.7
	B	39.3 ± 2.0	68.4 ± 3.4	94.1 ± 4.7
The initial concentration of PAL 50 mg				
I	A	33.2 ± 1.7	65.7 ± 3.3	87.0 ± 4.3
	B	31.8 ± 1.6	66.1 ± 3.3	88.3 ± 4.4
II	A	34.2 ± 1.7	66.3 ± 3.3	89.4 ± 4.5
	B	33.5 ± 1.7	67.8 ± 3.4	87.3 ± 4.4
III	A	36.7 ± 1.8	68.4 ± 3.4	90.2 ± 4.5
	B	35.5 ± 1.8	68.0 ± 3.4	88.6 ± 4.4
The initial concentration of PAL 100 mg				
I	A	45.6 ± 2.3	77.6 ± 3.9	96.8 ± 4.8
	B	47.2 ± 2.4	78.9 ± 3.9	95.7 ± 4.8
II	A	44.3 ± 2.2	79.0 ± 4.0	95.5 ± 4.8
	B	45.0 ± 2.3	78.3 ± 3.9	96.4 ± 4.8
III	A	46.6 ± 2.3	76.7 ± 3.8	96.4 ± 4.8
	B	48.1 ± 2.4	78.0 ± 3.9	96.7 ± 4.8

I—hepatoma Huh-7; II—monocytes ТНР-1; III—co-culture Huh-7 + ТНР-1; A—without capsules; B—with capsules. The experiments were carried out in triplicate.

**Table 2:** Results of determination of the content of phenylalanine metabolism products after incubation of model solutions with enzymes.

Incubation Duration, min	The Concentration of Phenylalanine Metabolic Products, μM														
	1	2	3	4	5	1	2	3	4	5	1	2	3	4	5
	PAH					Phenylalanine Transaminase					Encapsulated PAL				
activity 1.5 U/mg															
0	0.0 ± 0.0	− *	−	−	−	−	−	0.0 ± 0.0	0.0 ± 0.0	0.0 ± 0.0	−	0.0 ± 0.0	−	−	−
30	12.5 ± 0.6	−	−	−	−	−	−	10.6 ± 0.5	6.3 ± 0.3	7.8 ± 0.4	−	8.6 ± 0.4	−	−	−
60	23.4 ± 1.2	−	−	−	−	−	−	15.4 ± 0.8	9.5 ± 0.5	11.2 ± 0.6	−	15.0 ± 0.8	−	−	−
90	28.7 ± 1.4	−	−	−	−	−	−	19.0 ± 1.0	12.7 ± 0.6	14.5 ± 0.7	−	24.2 ± 1.2	−	−	−
120	33.2 ± 1.7	−	−	−	−	−	−	27.6 ± 1.4	15.2 ± 0.8	17.6 ± 0.9	−	31.5 ± 1.6	−	−	−
150	36.7 ± 1.8	−	−	−	−	−	−	34.5 ± 1.7	18.8 ± 0.9	21.3 ± 1.1	−	38.3 ± 1.9	−	−	−
180	41.2 ± 2.1	−	−	−	−	−	−	38.9 ± 1.9	21.0 ± 1.1	24.5 ± 1.2	−	46.7 ± 2.3	−	−	−
210	48.5 ± 2.4	−	−	−	−	−	−	42.1 ± 2.1	23.4 ± 1.2	27.7 ± 1.4	−	59.0 ± 3.0	−	−	−
240	56.0 ± 2.8	−	−	−	−	−	−	45.7 ± 2.3	25.3 ± 1.3	30.2 ± 1.5	−	77.8 ± 3.9	−	−	−
activity 3.0 U/mg															
0	0.0 ± 0.0	−	−	−	−	−	−	0.0 ± 0.0	0.0 ± 0.0	0.0 ± 0.0	−	0.0 ± 0.0	−	−	−
30	14.6 ± 0.7	−	−	−	−	−	−	13.3 ± 0.7	7.8 ± 0.4	10.5 ± 0.5	−	14.1 ± 0.7	−	−	−
60	27.0 ± 1.4	−	−	−	−	−	−	17.0 ± 0.9	11.0 ± 0.6	15.6 ± 0.8	−	22.5 ± 1.1	−	−	−
90	31.2 ± 1.6	−	−	−	−	−	−	21.3 ± 1.1	14.7 ± 0.7	17.2 ± 0.9	−	46.9 ± 2.3	−	−	−
120	36.7 ± 1.8	−	−	−	−	−	−	30.8 ± 1.5	17.3 ± 0.9	19.0 ± 1.0	−	62.8 ± 3.1	−	−	−
150	41.0 ± 2.1	−	−	−	−	−	−	37.8 ± 1.9	22.0 ± 1.1	24.4 ± 1.2	−	87.2 ± 4.4	−	−	−
180	52.0 ± 2.6	−	−	−	−	−	−	44.5 ± 2.2	26.7 ± 1.3	29.8 ± 1.5	−	95.3 ± 4.8	−	−	−
210	64.3 ± 3.2	−	−	−	−	−	−	57.0 ± 2.9	29.8 ± 1.5	35.1 ± 1.8	−	107.4 ± 5.4	−	−	−
240	75.8 ± 3.8	−	−	−	−	−	−	65.0 ± 3.3	32.1 ± 1.6	44.5 ± 2.2	−	116.0 ± 5.8	−	−	−
activity 5.0 U/mg															
0	0.0 ± 0.0	−	−	−	−	−	−	0.0 ± 0.0	0.0 ± 0.0	0.0 ± 0.0	−	0.0 ± 0.0	−	−	−
30	25.6 ± 1.3	−	−	−	−	−	−	16.0 ± 0.8	12.4 ± 0.6	13.6 ± 0.7	−	19.5 ± 1.0	−	−	−
60	48.9 ± 2.4	−	−	−	−	−	−	22.4 ± 1.1	17.8 ± 0.9	19.5 ± 1.0	−	30.8 ± 1.5	−	−	−
90	55.3 ± 2.8	−	−	−	−	−	−	25.6 ± 1.3	23.0 ± 1.2	25.6 ± 1.3	−	54.6 ± 2.7	−	−	−
120	60.7 ± 3.0	−	−	−	−	−	−	34.8 ± 1.7	26.9 ± 1.3	34.1 ± 1.7	−	68.2 ± 3.4	−	−	−
150	74.2 ± 3.7	−	−	−	−	−	−	40.6 ± 2.0	31.4 ± 1.6	39.2 ± 2.0	−	77.3 ± 3.9	−	−	−
180	88.0 ± 4.4	−	−	−	−	−	−	55.9 ± 2.8	36.2 ± 1.8	44.8 ± 2.2	−	95.2 ± 4.8	−	−	−
210	102.4 ± 5.1	−	−	−	−	−	−	67.4 ± 3.4	42.1 ± 2.1	47.9 ± 2.4	−	115.6 ± 5.8	−	−	−
240	118.7 ± 5.9	−	−	−	−	−	−	88.0 ± 4.4	48.7 ± 2.4	51.2 ± 2.6	−	138.2 ± 6.9	−	−	−

1—tyrosine; 2—trans-cinnamic acid; 3—phenylpyruvate; 4—phenyllactate; phenylacetate; * “−”—not found. The experiments were carried out in triplicate.

## Data Availability

Not applicable.
